# Deflux® Endoscopic Treatment of Vesicoureteral Reflux (VUR) in Japan

**DOI:** 10.3389/fped.2022.855378

**Published:** 2022-06-30

**Authors:** The Japanese Deflux Injection Therapy Study Group

**Affiliations:** Department of Pediatric General and Urogenital Surgery, Juntendo University School of Medicine, Bunkyo, Japan

**Keywords:** kidney, urinary tract infections, vesicoureteral reflux, Deflux®, endoscopic surgery

## Abstract

**Introduction:**

The aim of this study is to present the results of a multi-institutional outcome analysis conducted to determine the extent of endoscopic Deflux® injection for treating primary vesicoureteral reflux in Japan.

**Methods:**

A 22-question survey was distributed to 174 certified pediatric urologists (Ninteii in Japanese) and councilors of the Japanese Society of Pediatric Urology working at 140 centers to determine the usage and clinical efficacy of Deflux® for treating primary vesicoureteral reflux in Japan.

**Results:**

Forty-three of 140 (30.7%) centers participated, which exceeded participation rates of 27.9 and 18.0% for similar surveys conducted in America in 2006 and 2014, respectively. Deflux® was administered at 43 centers using subureteral transurethral injection (*n* = 19; 44.2%), hydrodistention implantation (*n* = 5; 11.6%), or double hydrodistention implantation (*n* = 19; 44.2%) and was the first-line treatment for primary vesicoureteral reflux at 39 (90.7%) centers. Overall, 1,563 ureters were treated in 1,076 patients. The male:female ratio was 527:549; mean follow-up was 5.1 years (range: 3.2–8 years); mean age at diagnosis of primary vesicoureteral reflux was 4.2 years, and mean age at first Deflux® treatment was 6.2 years. Overall cure rates were 65.3% after one Deflux® treatment, 75.3% after two, and 77.3% after three.

**Conclusion:**

To the best of our knowledge, this is the first multi-institutional outcome analysis of Deflux® usage for primary vesicoureteral reflux in Japan.

## Introduction

Vesicoureteral reflux (VUR) is one of the most common urologic diagnoses affecting children, with an estimated prevalence of ~1% in the general pediatric population and 30% in those with a history of febrile urinary tract infection (UTI) (1, 2). The diagnosis, symptoms, guideline, and history of VUR are described, elsewhere (3). Treatment options for managing VUR range from observation with or without continuous low-dose antibiotic prophylaxis to active surgical intervention with no consensus resulting in a variety of treatment protocols in active use around the world (4). The goals of treating a child with VUR are as follows: (1) to prevent recurring febrile UTI, (2) to prevent renal injury, and (3) to minimize the morbidity of treatment and follow-up (4).

The management of VUR was completely revolutionized by the first clinical report about an endoscopic procedure involving subureteric Teflon™ injection that was published in 1984 (5). This procedure was called subureteral transurethral injection (STING) and has since been modified to improve VUR cure rates, for example, by incorporating hydrodistention implantation (HIT) (6) or double HIT (7), and several tissue augmenting substances have been used for subureteral injection, such as polytetrafluoroethylene, collagen, silicone, autologous chondrocytes, and Deflux® (8), which have been used in place of Teflon™.

Deflux® consists of dextranomer microspheres of an average size of 80 to 250 μm in sodium hyaluronic acid solution (8). In 1995, the first clinical report about using Deflux® for grade III and IV VUR was published (9). The US Food and Drug Administration (FDA) approved the use of Deflux® for endoscopic injection in pediatric patients with primary VUR (pVUR) grades II–IV in 2001, and the frequency of endoscopic management of VUR has increased rapidly since then (1). In this study, VUR resolution was reported to be 68.3% 3 months after Deflux®.

Deflux® was officially approved for endoscopic injection in patients with pVUR grades II–IV by the Japanese Ministry of Health, Labor and Welfare in 2010, some 9 years after the FDA approved Deflux® in the United States. The contraindications for Deflux® use in Japan are presence of primary mega-ureter with distal stenosis, active UTI, nonfunctioning kidney, paraureteral diverticulum, or ureterocele.

Paraureteral diverticulum is a congenital lesion located at or adjacent to the ureteral hiatus where Waldeyer's sheath normally seals the potential space between the intravesical ureter and bladder muscle (10) that is associated with VUR.

Here, we present the results of a multi-institutional outcome analysis conducted to determine the extent of endoscopic Deflux® injection for treating pVUR in Japan and to focus on comparing the cure rate of Deflux® in Japan and other countries.

## Materials and Methods

The subjects for this study were pediatric patients with pVUR (aged <15 years old at the time of treatment) treated with Deflux® from the date of Deflux®'s approval for endoscopic injection in patients with pVUR grades II–IV by the Japanese Ministry of Health, Labor and Welfare to August 2016 and followed up more than 3 years. A 22-question survey (Supplementary Materials) was distributed to 174 certified pediatric urologists (Ninteii in Japanese) and councilors of the Japanese Society of Pediatric Urology working at 140 centers comprised of 62 university hospitals, 70 municipal/private hospitals, and eight clinics to determine the usage and clinical efficacy of Deflux® in Japan. The survey was distributed to each doctor as a Microsoft® Excel file and all completed surveys were collated and analyzed anonymously by the corresponding author (HM). For consistency, we defined “cure” after Deflux® as disappearance of VUR (grade 0) or downgrading to asymptomatic grade I on postoperative voiding cystourethrography (VCUG) performed 2 months after Deflux®.

### Statistical Analysis

Data were expressed as mean and range. The Fisher's exact test was used for categorical data and the Kruskal–Wallis test was used for continuous data (Prism 7; GraphPad Software, Inc., San Diego, CA, USA). Dunn's multiple comparisons test was used for multiple comparisons. A value of *p* < 0.05 was considered to be statistically significant.

## Results

Of the 140 treatment centers contacted, 54 agreed to participate in our multi-institutional outcome analysis. There were 27 university hospitals, 26 municipal/private hospitals, and one clinic. However, 11 of these centers were excluded because 10 did not use Deflux® and one center withdrew, leaving 43 centers (30.7%).

**Figure 1:**
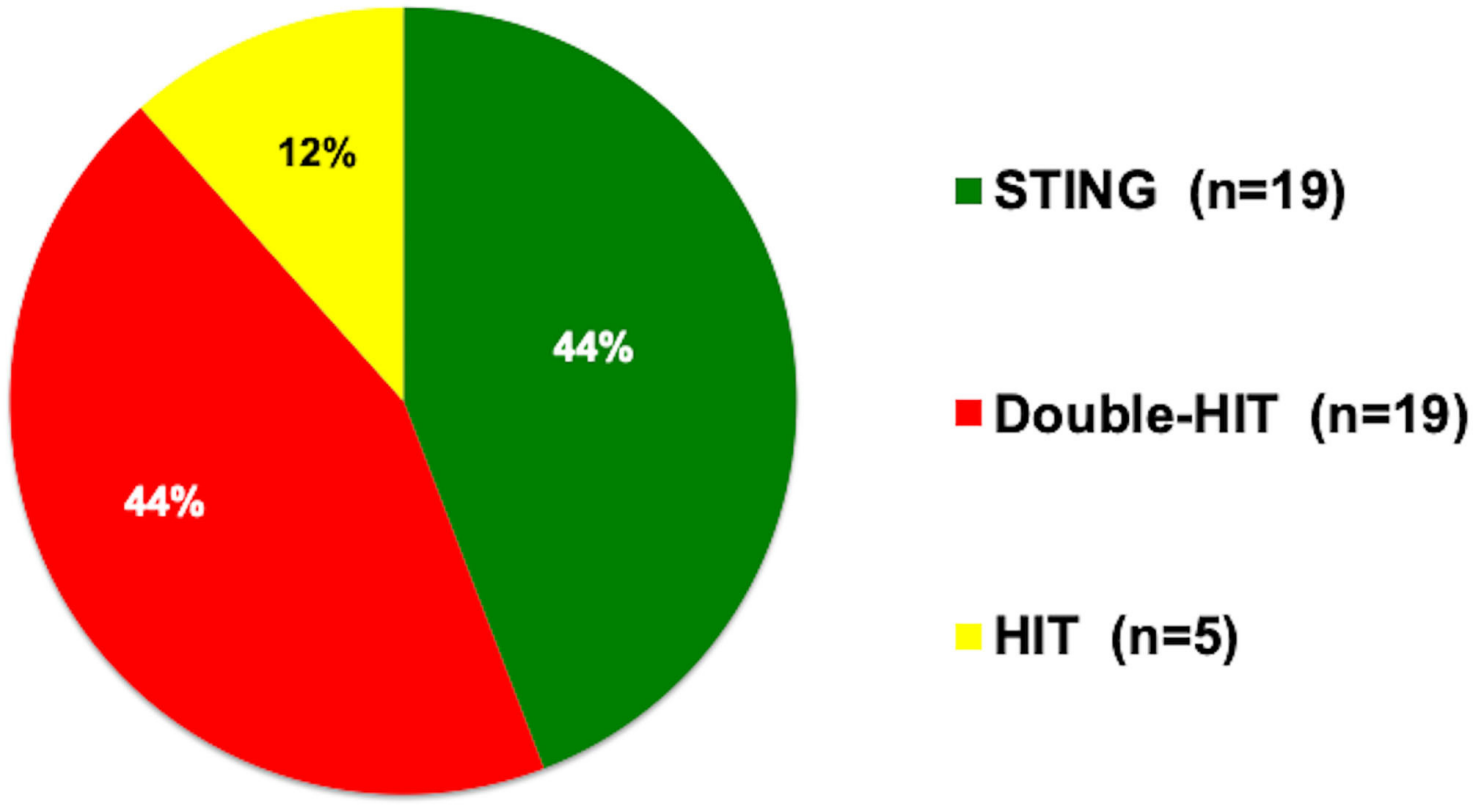
Preferred injection techniques. STING, subureteral transurethral injection; double HIT, double hydrodistention implantation; HIT, hydrodistention implantation.

First-line therapy for pVUR was Deflux® at 39 of 43 centers (90.7%), open surgery at two of 43 centers (4.7%), and endosurgery at two of 43 centers (4.7%). Techniques by which Deflux® was used were STING at 19 centers (44.2%), double HIT at 19 centers (44.2%), and HIT at five centers (11.6%) (Figure 1). When asked the indications for Deflux® as a “check all that apply” question, the top three responses were breakthrough UTI (*n* = 40 centers; 93.0%), presence of renal scarring (*n* = 31 centers; 72.1%), and parental request (*n* = 31 centers; 72.1%).

There were 1,563 ureters treated in 1,076 patients at the Deflux® centers: 487 bilateral, 264 right-sided, and 325 left-sided. Male:female ratio was 527:549. Fifty-three patients were diagnosed prenatally, usually with hydronephrosis, overall. Overall mean age at diagnosis of pVUR was 4.2 years. Mean age at the first Deflux® treatment was 6.2 years. Mean duration of follow-up from the first Deflux® was 5.1 years (range: 3.2–8 years).

UTI before Deflux® treatment was reported in 862 of 1,076 patients (80.1%). Of these, 385 reported one episode, 288 reported two episodes, 105 reported three episodes, and 84 reported more than four episodes. Renal scarring detected by 99mTc-DMSA (DMSA) scanning was reported in 481 kidneys before Deflux® treatment, but the incidence of DMSA scanning could not be determined.

From our multi-institutional outcome analysis, we found that 21 ureters associated with a paraureteral diverticulum had been treated with Deflux®. Similarly, although the endoscopic injection of Deflux® was only officially approved for treating VUR of grades II–IV, 12 centers (27.9%) also recorded treating grade V with Deflux®.

For all patients, the overall cure rate was 65.3% after one Deflux® treatment, 75.3% after two treatments, and 77.3% after three treatments (Table 1A). Overall cure rates after three treatments were not significantly different when results at 43 centers were compared by classifying the centers into three groups according to the number of ureters treated: 1–50 ureters, 51–99 ureters, and more than 100 ureters. For paraureteral diverticulum patients alone, cure rates were 42.9% after 1 Deflux® treatment, 52.4% after 2 treatments, and 52.4% after 3 treatments (Table 2A).

Table 1Resolution of vesicoureteral reflux (VUR) per ureter after Deflux®.

**Table:** 

	**First Deflux®**	**Second Deflux®**	**Third Deflux®**
**A**. Overall cure rates after one, two, and three Deflux® treatments.			
Overall Cure rate	958/1467^†^ (65.3%)	1,105/1,467^†^ (75.3%)	1134/1,467^†^ (77.3%)

**Table:** 

**VUR Grade**	**First Deflux**®	**Second Deflux**®	**Third Deflux**®	**Overall cure rate**
**B**. Overall cure rates according to VUR grades.				
II	252/347 (72.6%)	252 + 21/347 (78.7%)	273 + 2/347 (79.3%)	275/347 (79.3%)
III	438/658 (66.6%)	438 + 53/658 (74.6%)	491 + 11/658 (76.3%)	502/658 (76.3%)
IV	235/389 (60.4%)	235 + 56/389 (74.8%)	291 + 12/389 (77.9%)	303/389 (77.9%)
V	33/73 (45.2%)	33 + 17/73 (68.5%)	50 + 4/73 (74.0%)	54/73 (74.0%)

† *Excluding 96 grade I VUR ureters treated with Deflux®*.

Table 2Resolution of vesicoureteral reflux (VUR) per ureter after Deflux® (paraureteral diverticulum cases).

**Table:** 

	**First Deflux®**	**Second Deflux®**	**Third Deflux®**
**A**. Overall cure rates after one, two, and three Deflux® treatments.			
Overall Cure rate	9/21 (42.9%)	11/21 (52.4%)	11/21 (52.4%)

**Table:** 

**VUR Grade**	**First Deflux**®	**Second Deflux**®	**Third Deflux**®	**Overall cure rate**
**B**. Overall cure rates according to VUR grades.				
II	3/5 (60.0%)	3 + 0/5 (60.0%)	3 + 0/5 (60.0%)	3/5 (60.0%)
III	1/4 (25.0%)	1 + 0/4 (25.0%)	1 + 0/4 (25.0%)	1/4 (25.0%)
IV	5/7 (71.4%)	5 + 2/7 (100.0%)	7 + 0/7 (100.0%)	7/7 (100.0%)
V	0/5 (0%)	0/5 (0%)	0/5 (0%)	0/5 (0%)

Overall cure rates for all patients according to pre-Deflux® VUR grade were 79.3% for grade II, 76.3% for grade III, 77.9% for grade IV, and 74.0% for grade V (Table 1B). For paraureteral diverticulum patients alone, cure rates were 60.0% for grade II, 25.0% for grade III, 100.0% for grade IV, and 0% for grade V (Table 2B).

To summarize, cure rates when paraureteral diverticulum patients were not included were 65.6% after 1 Deflux® treatment, 75.7% after 2 treatments, and 77.7% after 3 treatments, and cure rates according to pre-Deflux® VUR grades when paraureteral diverticulum patients were not included were 79.5% for grade II, 76.6% of grade III, 77.5% of grade IV and 79.4% of grade V. The overall cure rate of grade V with paraureteral diverticulum was significantly lower than the overall cure rate of grade V without paraureteral diverticulum (*p* < 0.05), but the overall cure rates of grades II, III, and IV with paraureteral diverticulum were not significantly lower than the overall cure rates of grades II, III, and IV without paraureteral diverticulum.

Turning to complications, there were 112 ureters (7.2%) treated by open/endoscopic surgery after unsuccessful Deflux® at 20 centers. In response to whether previous Deflux® was an issue in any of these ureters, six centers reported that Deflux® was detrimental when treating 37 ureters, nine centers did not consider previous Deflux® relevant (29 ureters), and five centers were “unclear” (46 ureters). There were also 24 episodes of UTI within 1 year of Deflux® treatment, and 20 ureters in 18 patients had recurrence of VUR. Interestingly, there were 35 ureters diagnosed with *de novo* contralateral VUR after Deflux® treatment and 20 ureters requiring treatment. Ureteral obstruction developed in one ureter immediately after Deflux® and in one ureter a year after Deflux®. One was treated by insertion of a double J stent and the other resolved spontaneously during routine follow-up. Forty-one of 1,076 (3.8%) patients complained of transient flank pain after Deflux®.

For follow-up, only 32 of 43 centers (74.4%) performed VCUG 1 year after Deflux® routinely.

## Discussion

From our multi-institutional outcome analysis, we found that the overall cure rate after three Deflux® treatments was 77.3%. A systematic review of 47 Deflux® studies concluded that there was 77% success rate per ureter (11). We tried analyzing success rates with respect to experience by categorizing participating centers according to the number of ureters treated; i.e., 1–50 ureters (*n* = 33), vs. 51–99 ureters (*n* = 4), vs. 100 or more (*n* = 6), but we found no significant differences in outcome based on experience. Unfortunately, we could not investigate this further because it was beyond the scope of our study, because we did not define how to count ureters that may have had multiple treatments, for example, when Deflux® was attempted but failed, and the patient had open surgery.

According to the American Urological Association guidelines published in 2010, the rationale for recommending curative therapy for VUR associated with breakthrough UTI was prevention of renal injury (4), and we found that breakthrough UTI was the most common indication for Deflux® use chosen from the standard indications offered in the survey (breakthrough UTI, progressive renal scarring, noncompliance with medical therapy, nonresolution of VUR, and parental request) (12).

While we collected information about preferred injection techniques, our results were so completely different that no comparison could be made. For example, the most commonly preferred techniques in Japan were tied between STING and double HIT at 44.2%, in stark contrast to the United States where double HIT was performed by 92.0% (1).

VUR in paraureteral diverticulum cases is not generally considered as being primary. Cerwinka reported that they achieved a success rate of 81.3% in treating VUR associated with paraureteral diverticulum with Deflux®, despite leaving the paraureteral diverticula behind in half of their subjects (13). From our multi-institutional outcome analysis, 11 of 43 centers (25.6%) reported using Deflux® for treating VUR associated with paraureteral diverticulum. The overall cure rate was 42.9, 52.4, and 52.4% after one, two, and three Deflux® treatments, which would indicate that there may be some limitation to the effectiveness of Deflux® in certain circumstances or the proportion of grade V cases was higher in our results so our results were worse than Cerwinka's.

Repeat VCUG 1 year after Deflux® is thus a valuable investigation, but, from our multi-institutional outcome analysis, we found that it was performed at 32 of 43 centers (74.4%). In 2009, Lee reported the outcome of Deflux® after 1 year in children in whom VUR was noted to have resolved on postoperative VCUG. VUR resolution was 73% initially but, when repeated 1 year later, had fallen to 46.1% (14) probably as a consequence of natural migration of injected Deflux® over time, which could be implicated as a potential cause of recurrence at some time in the future. Longer follow-up studies with “follow-up” defined universally will be required to confirm the efficacy of Deflux®.

### Limitations

Some limitations exist in this study. First, we did not measure the volumes of Deflux® injected and we did not define “cure” and “follow-up” specifically enough. Second, we could not compare our data with existing data because there was such a broad spectrum of “cure” used in reports in the literature, ranging from no VUR on postoperative imaging to successful downgrading if the final VUR is less severe than the original (11). Third, we did not ask the centers about the reason why the patients who had no previous UTI were diagnosed pVUR. Fourth, regarding *de novo* contralateral VUR, we did not ask the centers whether they routinely injected Deflux® into the contralateral side, based on its appearance with hydrodistention. Fifth, we did not ask the centers about the circumcision status of boys. Finally, we did not ask the centers about the characteristics of the ureteral obstruction cases after Deflux® treatment.

Even based on the items stated in our limitation section, as the 140 target centers of the survey sheet are not restricted to those using Deflux® treatment, we have received the response from over 30%, and that reflects the same rate of successful Deflux® treatments on 1,563 ureters as the systematic review.

## Author's Note

The Japanese Deflux® Injection Therapy Study Group (Appendix A). All doctors listed in Appendix A contributed equally to this work.

## Data Availability Statement

The original contributions presented in the study are included in the article/Supplementary Material, further inquiries can be directed to the corresponding author.

## Ethics Statement

This study was approved by the Ethics Committee of all centers (No. 15-018: Juntendo University School of Medicine). The Ethics Committee waived the requirement of written informed consent for participation.

## Author Contributions

The author listed in the Appendix A were involved in clinical treatment, collected and analyzed the data, and contributed to the article and approved the submitted version.

## Conflict of Interest

The author declares that the research was conducted in the absence of any commercial or financial relationships that could be construed as a potential conflict of interest.

## Publisher's Note

All claims expressed in this article are solely those of the authors and do not necessarily represent those of their affiliated organizations, or those of the publisher, the editors and the reviewers. Any product that may be evaluated in this article, or claim that may be made by its manufacturer, is not guaranteed or endorsed by the publisher.

## Appendix

**Appendix A:** The japanese deflux® injection therapy study group.

**Institute**	**Name**
Aichi Children's Health and Medical Center	Masato Watanabe, Kaoru Yoshino
Chukyo Hospital	Yoshikazu Tsuji
Department of Renal and Genitourinary Surgery, Hokkaido University	Kimihiko Moriya
Department of Pediatric Surgery, Faculty of Medical Sciences, Kyusyu University	Yoshiaki Kinoshita, Tomoaki Taguchi
Department of Pediatric Surgery, Nihon University School of Medicine	Takeshi Furuya, Takayuki Masuko
Department of Urology, Ehime University Graduate School of Medicine	Kenichi Nishimura
Department of Urology, Faculty of Medicine, University of Miyazaki	Toshio Kamimura
Department of Urology, Graduate School of Medicine, University of the Ryukyus	Minoru Miyazato
Department of Urology, Kagoshima University Graduate School of Medical and Dental Sciences	Toshihiko Itesako
Department of Urology, Kanagawa Children's Medical Center	Takashi Ikeda, Yuichiro Yamazaki
Department of Urology, Kyoto Prefectural University of Medicine	Yasuyuki Naito, Yasuhiro Yamada
Department of Urology, Oita University Faculty of Medicine, Oita, Japan	Yasuyuki Akita, Kenichi Mori
Department of Urology, Osaka Women's and Children's Hospital	Fumi Matsumoto
Department of Urology, Shiga University of Medical Science	Kazuyoshi Johnin, Akihiro Kawauchi, Kenichi Kobayashi
Division of Urology, Niigata University Graduate School of Medical and Dental Sciences	Sayaka Akiyama, Kenji Obara
Hiroshima City Hiroshima Citizens Hospital	Takashi Akiyama
Hyogo Prefectural Kobe Children's Hospital	Yoshifumi Sugita
Ibaraki Children's Hospital	Toshihiro Yanai
Iwakuni Medical Center	Morimichi Tani
Japanese Red Cross Akita Hospital	Takashi Obara
Juntendo University Nerima Hospital	Takaaki Imaizumi, Masahiko Urao
Juntendo University Urayasu Hospital	Yuki Ogasawara
Kanazawa Medical University	Miyuki Kohno
Kindai University Nara Hospital	Katsuji Yamauchi, Takeo Yonekura
Kochi Health Sciences Center	Kiyoshi Sasaki
Kurashiki Medical Center	Takaharu Ichikawa
Maternal and Children's Health Center Aiiku Hospital	Takao Fujimoto
Mitsui Hospital	Shuji Sugimoto
Moriguchi Ikuno Memorial Hospital	Akira Tohda
Nagano Children's Hospital	Midori Ichino
Okayama Medical Center	Takafumi Goto
Okayama Rosai Hospital	Yoshitsugu Nasu
Osaka City General Hospital	Wataru Sakamoto
Osaka City University Medical School, Pediatric Surgery	Yoshiki Morotomi
Pediatric General and Urogenital Surgery, Juntendo University School of Medicine	Hiroshi Murakami, Manabu Okawada, Atsuyuki Yamataka
Pediatric Surgery Department, Okayama Univ. Hospital	Takuo Noda
Saitama Children's Medical Center	Yutaro Hori, Kensuke Ohashi
Shikoku Medical Center for Children and Adults	Yoshinobu Iwamura
Tokai University Oiso Hospital	Hideshi Miyakita
Tokyo Medical University, Department of Gastrointestinal and Pediatric Surgery	Yutaka Hayashi
Tokyo Metropolitan Children's Medical Center, Department of Urology and Kidney Transplants	Hiroyuki Satoh
Tokyo Women's Medical University, Department of Pediatric Surgery	Osamu Segawa, Ryusuke Yamaguchi
Tokyo Women's Medical University, Department of Urology, Kidney Center	Rie Yago
