# Supplementary material for: Deflux® Endoscopic Treatment of Vesicoureteral Reflux (VUR) in Japan
Source: Front Pediatr. 2022 Jun 30;10:855378. doi: 10.3389/fped.2022.855378 (PMC10157820; doi:10.3389/fped.2022.855378)
Supplement: Supplementary file 1 [file Data_Sheet_1.docx]

**Appendix B: Survey instrument**

1. Have you ever administered Deflux^®^ for primary VUR (pVUR)?

1. YES
2. NO (No further questions)

2. In your practice do you administer Deflux^®^ or perform open surgery or endoscopic surgery as first-line therapy for pVUR? (Choose one response).

1. Open surgery (Cohen procedure or Politano-Leadbetter procedure or other)
2. Deflux^®^
3. Endoscopic surgery
4. Open surgery and Deflux^®^
5. Open surgery and endoscopic surgery
6. Deflux^®^ and endoscopic surgery
7. Open surgery, Deflux^®^ and endoscopic surgery

3. Which of the following techniques do you prefer to use for administering Deflux^®^?

1. STING (conventional)
2. HIT
3. Double- HIT

4. What are your indications for Deflux^®^? (Check all that apply).

1. Breakthrough UTI
2. UTI (1 time)
3. Renal scarring
4. Parental request
5. VUR grade ≥ II
6. Other

5. Please input your data for frequency of UTI before 1^st^ Deflux^®^

1 episode: INPUT patients

2 episodes: INPUT patients

3 episodes: INPUT patients

4 or more episodes: INPUT patients

6. Please input your data for location of VUR treated by Deflux^®^.

Right VUR: INPUT patients

Left VUR: INPUT patients

Bilateral VUR: INPUT patients

Fetal diagnosis: INPUT patients

7. Please input your data for mean age at diagnosis of pVUR.

INPUT years

8. Please input your data for mean age at 1^st^ Deflux^®^.

INPUT years

9. Please input your data for mean duration of follow-up after 1^st^ Deflux^®^ including the range.

INPUT years

10. Please input your data for sex of patients treated by Deflux^®.^

Male: INPUT , Female: INPUT

11. Please input your data for preoperative VUR grades.

Grade I: INPUT ureters

Grade II: INPUT ureters

Grade III: INPUT ureters

Grade IV: INPUT ureters

Grade V: INPUT ureters

12. Please input your data for resolution of VUR after Deflux^®^.

(Definition of cure: VUR grade≤ I after Deflux^®^)

| Preoperative VUR grade | 1^st^ Deflux^®^ | 2^nd^ Deflux^®^ | 3^rd^ Deflux^®^ | Persistent |
| --- | --- | --- | --- | --- |
| II | INPUT (ureters) | INPUT (ureters) | INPUT (ureters) | INPUT (ureters) |
| III | INPUT (ureters) | INPUT (ureters) | INPUT (ureters) | INPUT (ureters) |
| IV | INPUT (ureters) | INPUT (ureters) | INPUT (ureters) | INPUT (ureters) |
| V | INPUT (ureters) | INPUT (ureters) | INPUT (ureters) | INPUT (ureters) |

13. (FOR CASES ASSOCIATED WITH PARAURETERAL DIVERTICULUM ONLY)

Please input your data for preoperative pVUR grades.

Grade I: INPUT ureters

Grade II: INPUT ureters

Grade III: INPUT ureters

Grade IV: INPUT ureters

Grade V: INPUT ureters

14. (FOR CASES ASSOCIATED WITH PARAURETERAL DIVERTICULUM ONLY)

Please input your data for resolution of VUR after Deflux^®^.

(Definition of cure: VUR grade≤ I after Deflux^®^)

| Preoperative VUR grade | 1^st^ Deflux^®^ | 2^nd^ Deflux^®^ | 3^rd^ Deflux^®^ | Persistent |
| --- | --- | --- | --- | --- |
| II | INPUT (ureters) | INPUT (ureters) | INPUT (ureters) | INPUT (ureters) |
| III | INPUT (ureters) | INPUT (ureters) | INPUT (ureters) | INPUT (ureters) |
| IV | INPUT (ureters) | INPUT (ureters) | INPUT (ureters) | INPUT (ureters) |
| V | INPUT (ureters) | INPUT (ureters) | INPUT (ureters) | INPUT (ureters) |

15. Please input your data for the number of ureters requiring open surgery or endoscopic surgery after Deflux^®^

Was unsuccessful Deflux^®^ detrimental to Open/endoscopic surgery?

1. YES
2. NO
3. Unclear

16. Please input your data for the incidence of ureteral obstruction after Deflux^®^.

How did you treat postoperative ureteral obstruction?

1. Double J Stent
2. Urinary diversion
3. Other (please list)

17. Please input your data for postoperative UTI within 1 year of Deflux^®^.

INPUT patients

18. Please input your data for recurrence of VUR in the patients in Q17.

INPUT patients (INPUT ureters)

19. Please input your data for the incidence of postoperative *de novo* contralateral VUR and how many required surgical interventions.

INPUT ureters (INPUT required surgical intervention)

20. Please input your data for the incidence of flank pain after administration of Deflux^®^.

INPUT patients

21. Please input your data for the incidence of preoperative renal scarring of kidneys associated with VUR.

INPUT kidneys

22. Do you perform repeat VCUG 1 year after Deflux^®^?

1. YES
2. NO
